# Functional analysis of the *Arabidopsis thalianaMUTE* promoter reveals a regulatory region sufficient for stomatal-lineage expression

**DOI:** 10.1007/s00425-015-2445-7

**Published:** 2016-01-09

**Authors:** Aaron K. Mahoney, Elizabeth M. Anderson, Rachael A. Bakker, Anthony F. Williams, Jake J. Flood, Katrina C. Sullivan, Lynn J. Pillitteri

**Affiliations:** Department of Biology, Western Washington University, Bellingham, WA 98225, USA

**Keywords:** *Arabidopsis*, *Cis*-element, Meristemoid, *MUTE*, Promoter, Stomata

## Abstract

The *MUTE* promoter contains a 175-bp region rich in Dof regulatory elements (AAAG) that is necessary and sufficient for initiation of transcription in meristemoids and the stomatal lineage.

The molecular mechanism underlying the decision to divide or differentiate is a central question in developmental biology. During stomatal development, expression of the master regulator *MUTE* triggers the differentiation of meristemoids into stomata. In this study, we carried out *MUTE* promoter deletion analysis to define a regulatory region that promotes the initiation of expression in meristemoids. Expression constructs with truncated promoter fragments fused to β-glucuronidase (GUS) were developed. The full-length promoter and promoter truncations of at least 500 bp from the translational start site exhibited normal spatiotemporal expression patterns. Further truncation revealed a 175-bp promoter fragment that was necessary and sufficient for stomatal-lineage expression. Known *cis*-elements were identified and tested for functional relevance. Comparison of orthologous *MUTE* promoters suggested DNA binding with one finger (Dof) regulatory elements and novel motifs may be important for regulation. Our data highlight the complexity and combinatorial control of gene regulation and provides tools to further investigate the genetic control of stomatal development.

**Electronic supplementary material:**

The online version of this article (doi:10.1007/s00425-015-2445-7) contains supplementary material, which is available to authorized users.

## Introduction

**Fig. 1:**
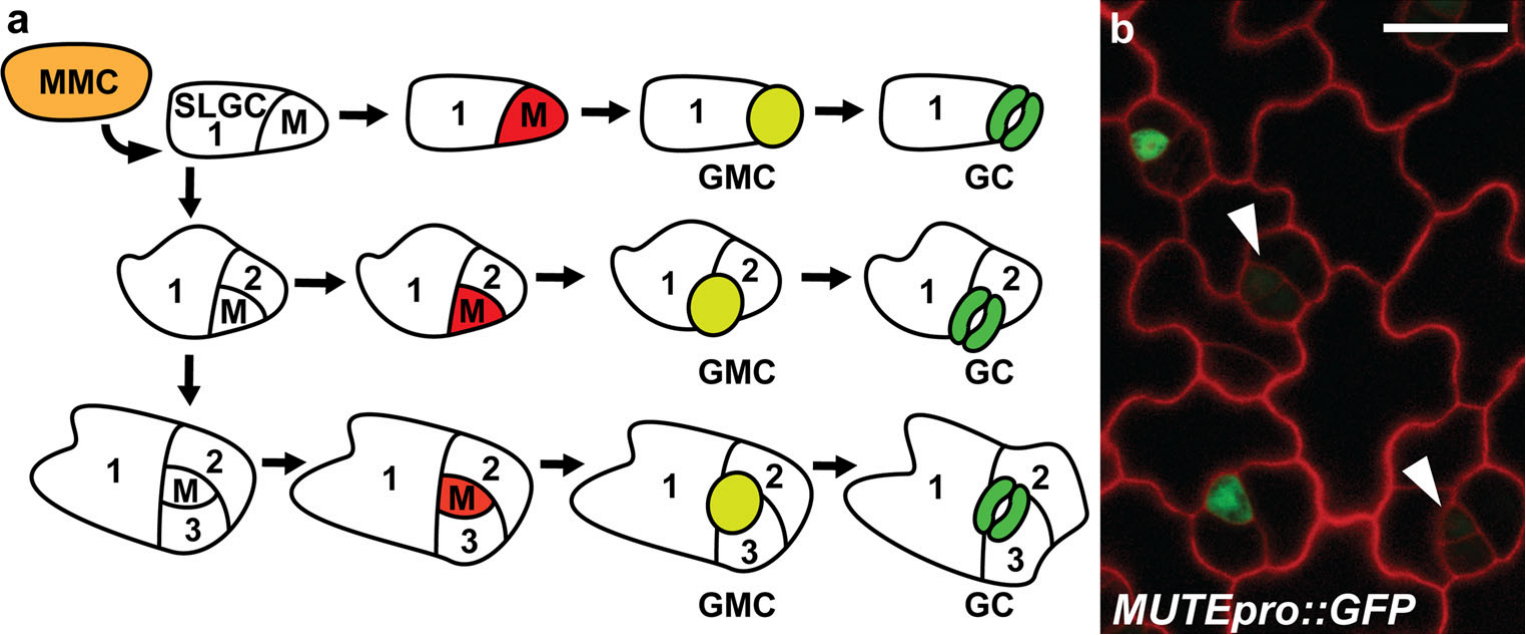
Diagram of division plasticity in the stomatal lineage. **a** A meristemoid mother cell (*MMC*, *orange*) divides asymmetrically to enter the stomatal lineage and creates a smaller meristemoid (*M*) and a stomatal lineage ground cell (*SLGC*). Meristemoids proceed through a variable number of asymmetric divisions and transition into a guard mother cell (*GMC*, *yellow*) based on the expression of *MUTE* (*red*). A GMC divides once symmetrically to produce two equally sized guard cells (*GC*, *green*) which are terminally differentiated. **b** Full-length *MUTE* promoter driving GFP expression in 12-day-old seedling abaxial leaf epidermis. The *MUTE* promoter is not active in meristemoids that will continue to divide (*arrowhead*s). *Scale bar* 10 µm

Stem cells are essential for the production of cellular and tissue diversity across all organisms. Stem cells have the ability to divide asymmetrically to produce two daughter cells with different fates; one cell retains stem-cell characteristics, while the other may differentiate (Aichinger et al. 2012). In plants, several cell types outside major stem-cell niches have permanent or transient stem-cell characteristics (Fisher and Turner 2007; Pillitteri et al. 2007; Miyashima et al. 2013). Stomatal meristemoids are one such cell type. Stomata are epidermal structures composed of two guard cells that surround an intervening pore, where the size of the pore can be regulated based on turgor-driven changes in each guard cell. In dicotyledonous plants, the production of a stoma initiates with an unequal division of a meristemoid mother cell (MMC), which produces a meristemoid and a larger sister cell called a stomatal lineage ground cell (SLGC) (Fig. 1a). The SLGC can differentiate into a lobed epidermal cell or divide again to produce an additional meristemoid. A meristemoid can self-renew through repeated divisions (up to 3 additional times) or differentiate into a guard mother cell (GMC). The GMC undergoes one symmetric division to produce two terminally differentiated guard cells (Vaten and Bergmann 2012; Pillitteri and Dong 2013).

The number of times a meristemoid divides prior to GMC differentiation is a plastic trait regulated by internal and external factors. Research over the past decade has revealed a prominent role of basic helix–loop–helix (bHLH) proteins in the differentiation of stomatal-lineage cell types. One of these proteins, *MUTE*, is required for the differentiation of meristemoids (Pillitteri et al. 2007). Loss-of-function *MUTE* plants are stomataless and produce meristemoids that continually divide without differentiation. Conversely, ectopic overexpression of *MUTE* results in the conversion of all epidermal cells into stomata. Consistent with its function, the promoter activity of *MUTE* is only observed in the stomatal lineage, where expression is activated in a subset of meristemoids that will differentiate into stomata (Fig. 1a, b) (Pillitteri et al. 2008). A close paralog of *MUTE* called *SPEECHLESS* (*SPCH*) has also been implicated in meristemoid differentiation. Weak *spch* mutants produce meristemoids that undergo fewer divisions and prematurely differentiate into a GMC (MacAlister et al. 2007; Lau et al. 2014). Two additional bHLH proteins, SCREAM (SCRM) and its paralog SCRM2 physically interact with MUTE and SPCH to regulate their respective functions (Kanaoka et al. 2008).

Investigation into the plasticity of meristemoid divisions led to the identification of several signal transduction genes that promote or inhibit stomatal production (Vaten and Bergmann 2012; Pillitteri and Dong 2013). For example, loss-of-function mutations in some ERECTA-family (ERf) leucine-rich repeat receptor-like kinases (LRR-RLK) result in a reduction in the number of meristemoid divisions prior to GMC differentiation (Shpak et al. 2005). Similarly, alterations in the expression of the LRR receptor-like protein, TOO MANY MOUTHS (TMM), the subtilisin protease, STOMATAL DENSITY AND DISTRIBUTION1 (SDD1), small peptide ligands, or several members of the YODA (YDA) MAP kinase cascade result in fewer meristemoid divisions than wild type (Berger and Altmann 2000; Geisler et al. 2000; Hara et al. 2007; Lampard et al. 2008; Hunt and Gray 2009; Gudesblat et al. 2012; Jewaria et al. 2013). These signaling components are thought to mediate hormonal and environmental signals to direct stomata production under diverse conditions (Kim et al. 2012; Balcerowicz et al. 2014). Direct links between these signaling pathways and *SPCH* expression have been demonstrated (Lampard et al. 2008; Gudesblat et al. 2012).

The molecular basis of expression of some stomatal regulatory components has been investigated and broad microarray analyses have revealed novel stomatal-lineage specific genes and potential DNA binding sites for some stomatal regulators (Chinnusamy et al. 2003; Lai et al. 2005; Ohashi-Ito and Bergmann 2006; Pillitteri et al. 2011; Hachez et al. 2011; Adrian et al. 2015). Transcriptional profiling of meristemoids provided insight into the broad characteristics of meristemoids (Pillitteri et al. 2011; Adrian et al. 2015). However, detailed information on the transcriptional elements that control gene activation in this cell type is lacking. Because meristemoids are capable of reiterative asymmetric cell division, they are useful tools for investigating the molecular basis of stem-cell character. This work reports a detailed characterization of the promoter region of *MUTE*. Deletion analysis of the *MUTE* promoter led to the identification of a small 175-bp region that is necessary and sufficient to drive stomatal-lineage specific expression. In silico analysis revealed the presence of conserved DNA binding with one finger (Dof) binding elements within this region in orthologous genes, suggesting they may be important for regulation. Together, these findings further our understanding of the molecular mechanisms involved in gene regulation and provide tools to identify specific proteins that bind the *MUTE* promoter and regulate the differentiation of meristemoids.

## Materials and methods

### Plant transformation and plant growth conditions

*Arabidopsis thaliana* ecotype Columbia (Col) were used for transformation. Plants were grown under a 16 h/8 h light/dark cycle at 22–24 °C. All transformation constructs were transferred to *Agrobacterium tumefaciens* strain GV3101 by electroporation. Transformation of *Arabidopsis* was done by *Agrobacterium*-mediated transformation using floral dip method (Clough and Bent 1998). Seeds from all lines were surface sterilized with 30 % (v/v) bleach for 10 min. Transformants were selected by plating seeds on 0.5 MS media containing the appropriate antibiotic for plant selection and timentin (50 µg/ml). Positive transformants were confirmed by polymerase chain reaction (PCR) genotyping. Homozygous lines were confirmed by segregation ratio and used for analysis.

### 5′ and 3′-rapid amplification of cDNA ends (RACE)

Total RNA was extracted from 10-day-old Col seedlings using Qiagen RNeasy kit according to manufacturer’s instructions. 1 µg of total RNA was subjected to 5′ RACE with the GeneRacer Kit (Invitrogen) according to manufacturer’s instructions. Briefly, total RNA was dephosphorylated with calf intestinal phosphatase to eliminate RNAs that were not full length. Dephosphorylation with tobacco acid pyrophosphatase was used to remove the cap structure of full-length mRNAs and the GeneRacer RNA oligo was ligated to the 5′ end of the transcript. The RNA was reverse transcribed using the GeneRacer oligo dT primer and Superscript II Reverse Transcriptase. Nested PCR was used for both 5′ and 3′ end amplification. Nested PCR conditions used for amplification were as follows: (96 °C, 2 min); 5 cycles at (96 °C, 20 s; 72 °C, 30 s; 72 °C, 30 s); 5 cycles at (95 °C, 20 s; 70 °C, 30 s; 72 °C, 30 s) and 23 cycles at (95 °C, 20 s; 68 °C, 30 s; 72 °C, 30 s) using the standard primers from the kit and gene-specific primers listed in Suppl. Table S1. Amplified products were cloned into TOPO pCR4 TA cloning vector according to manufacturer’s instructions and sequenced using an M13F primer.

### Vector construction

The full-length *MUTE* promoter designated in this study includes 1954 bp of promoter sequence immediately upstream of the translation initiation codon (Pillitteri et al. 2007). A series of progressive 5′-flanking region deletion fragments of the full-length promoter was generated by PCR using promoter-specific primers (Suppl. Table S2). Forward and reverse primers were designed with *Eco*RI sites and all amplification products were digested with *Eco*RI and cloned directly into *Eco*RI-digested LJP138, which contains an *Eco*RI site directly upstream of the GUS reporter gene (Pillitteri et al. 2007).

For promoter complementation using the 175-bp fragment, primers were designed to amplify −500 to −325 bp of the *MUTE* promoter. The amplicon was blunt-cloned into LJP312 containing the 35S CaMV minimal promoter element from −46 to +8 bp (Bhullar et al. 2007). For the second PCR reaction, primers were designed to with *Eco*RI sites. The amplicon included the −500 to −325-bp fragment fused to the 35S CaMV minimal promoter. This amplicon was digested with *Eco*RI and cloned directly into *Eco*RI-digested LJP138. For the 86 bp fragment, complementary oligos were designed that contained the *MUTE* promoter sequence from −411 to −325-bp upstream of the 35S CaMV minimal promoter The oligos were designed to produce *Eco*RI overhangs when hybridized (Suppl. Table S3). Oligos were resuspended at the same molar concentration in annealing buffer (10 mM Tris pH 8.0, 50 mM NaCl, 1 mM EDTA). For annealing, oligos were combined in equimolar amounts, heated to 95 °C for 5 min followed by slow cooling (1 °C/min) to reach room temperature. Annealed oligos were used directly in a ligation reaction with *Eco*RI-digested LJP138.

### Site-directed mutagenesis

Two independent PCR reactions were performed for each mutagenesis reaction (Heckman and Pease 2007) using primers in Suppl. Table S4. Final amplification products containing the mutagenized *cis*-element were gel-purified using an UltraClean PCR Clean-Up Kit (MO BIO Laboratories, Inc.) and cloned into TOPO pENTR (Invitrogen) according to manufacturer’s instructions. To produce GFP reporter constructs, vectors were combined with pGWB4 destination vector (Nakagawa et al. 2007) containing the recombination sites upstream of the coding sequence of the GFP using LR Clonase (Invitrogen).

### Histochemical staining and microscopy

Histochemical analysis of GUS activity was performed in GUS reaction buffer (0.5 mg/ml 5-bromo-4-chloro-3-indolyl-beta-d-glucuronide in 100 mM sodium phosphate, pH 7.0) as described previously (Pillitteri et al. 2008). GUS-stained samples were taken through an ethanol series to remove chlorophyll and placed in chloral hydrate solution (1:8:1, by vol., glycerol:chloral hydrate:water) for 1 h before imaging. All samples were viewed under DIC optics using a Leica Leitz DMRB microscope (Buffalo Grove, IL, USA) equipped with SPOT RT3 Cooled CCD camera (Sterling Heights, MI, USA). Confocal images were taken on an Olympus FV1000 (Center Valley, PA, USA). Cell borders were visualized with propidium iodide. Confocal images were false colored and brightness/contrast setters were adjusted using Photoshop CS4.

### Analysis of promoter sequences

Regulatory elements in the *MUTE* promoter were analyzed using the online program PLACE (a database of plant *cis*-acting regulatory DNA elements) and PlantCARE (plant *cis*-acting regulatory elements, enhancers and repressors). These two programs are available at http://www.dna.affrc.go.jp/PLACE/ and http://bioinformatics.psb.ugent.be/webtools/plantcare/html/, respectively. Alignment of orthologous promoters was performed using Clustal Omega with manual adjustments.

## Results

### Promoter and transcript analysis of *MUTE*

**Fig. 2:**
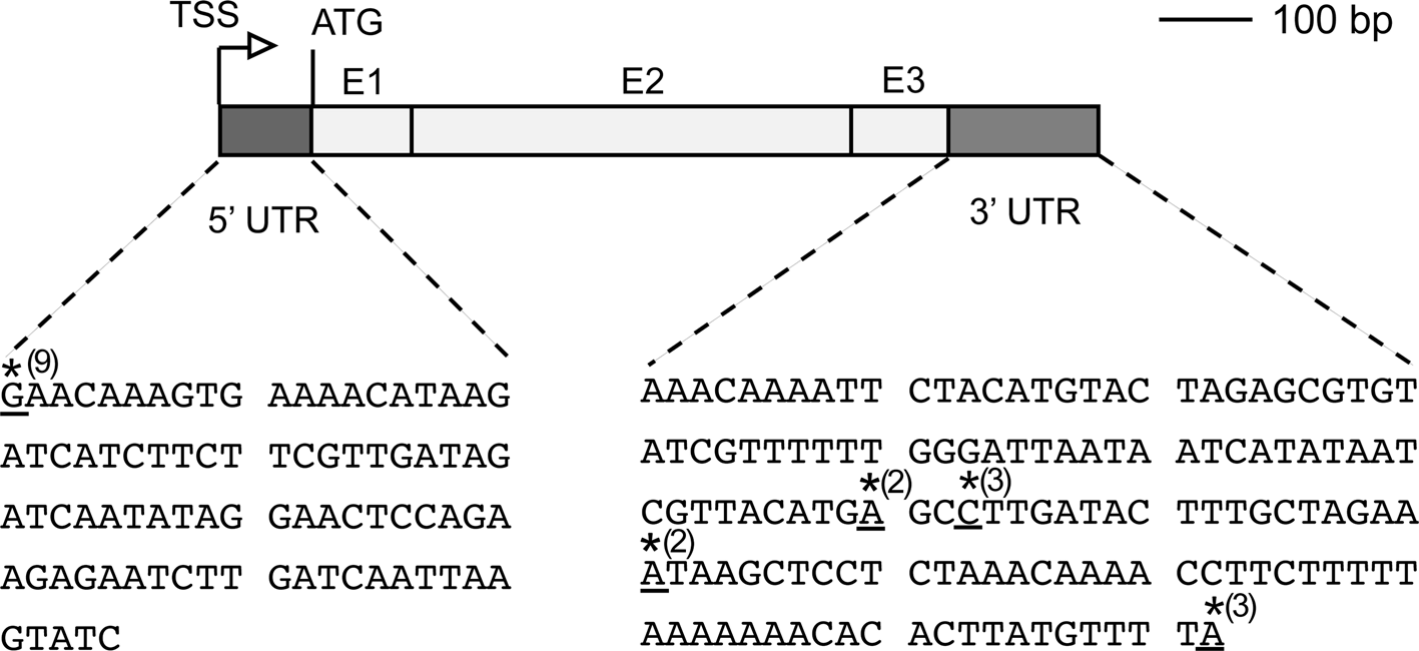
Diagram depicting the 5′ and 3′ untranslated regions (*UTRs*) of *MUTE*. The *MUTE* 5′ transcriptional start site (*TSS*), indicated by *underline* and *asterisk*, is 85 bp upstream of the start codon (*ATG*) (*n* = 9). The polyadenylation site was heterogeneous. The multiple 3′ polyadenylation sites are indicated with *underline* and *asterisk*. The *number in parenthesis* indicates the number of clones that ended at that position (*n* = 10 total). *Grey boxes* represent UTRs, *light grey boxes* indicate exons (E1, E2 and E3)

Untranslated regions (UTRs) of mRNA transcripts are important contributors to transcript stability, spatial and temporal expression patterns, and translation efficiency (Molina and Grotewold 2005; Srivastava et al. 2014; Kim et al. 2014). Alternate transcriptional start sites (TSS) and polyadenylation can produce diversity that further affects mRNA transport or stability to impact gene expression (Shen et al. 2008). Given the transient and meristemoid-specific expression of *MUTE*, we were interested in accurately mapping the TSS and polyadenylation sites of the *MUTE* transcript. To this end, we performed 5′ and 3′ RACE (rapid amplification of cDNA ends). Our results indicate that *MUTE* has an invariable TSS (*n* = 9) 29 bp downstream of the TATA-box consensus sequence (TATAAAT). The 5′ UTR is 85 bp in length, which is consistent with the trend for short 5′ UTRs in TATA-box-containing promoters in *Arabidopsis* (Kawaguchi and Bailey-Serres 2005; Molina and Grotewold 2005) (Fig. 2).

In contrast to the uniform TSS, *MUTE* has multiple polyadenylation sites clustered in close proximity to one another (Fig. 2). The range of 3′ UTR size was between 70 and 142 bp, which is smaller than the average 3′ UTR in *Arabidopsis* (Shen et al. 2008). Variation in 3′ UTR length is common and several studies have determined that cell proliferation or developmental stage can cause a shift in polyadenylation sites (Proudfoot 2011). Messenger RNAs with longer 3′ UTRs have more potential for miRNA binding that can impact stability under specific developmental states (Sandberg et al. 2008; Proudfoot 2011). We found no evidence of miRNA binding sites within the 3′ UTR or anywhere in the *MUTE* coding region using publicly available data, suggesting that *MUTE* expression is not regulated by known miRNAs (Zhang et al. 2010).

### Delineation of sequence elements required for stomatal-lineage-specific expression

**Fig. 3:**
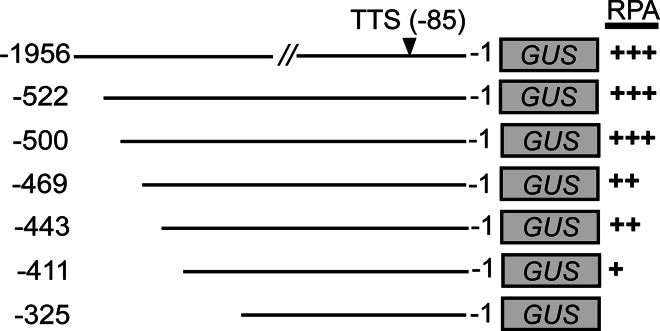
*MUTE* full-length promoter and deletion derivatives. *Solid lines* indicate promoter length; *grey box* indicates *GUS* coding sequence. *Numbers* indicate the nucleotide position relative to the *MUTE* translation start site (+1) for each construct. Transcriptional start site (*TSS*) is indicated. Relative promoter activity (*RPA*) is given (+, ++, +++) based on qualitative comparison to the full-length promoter

**Fig. 4:**
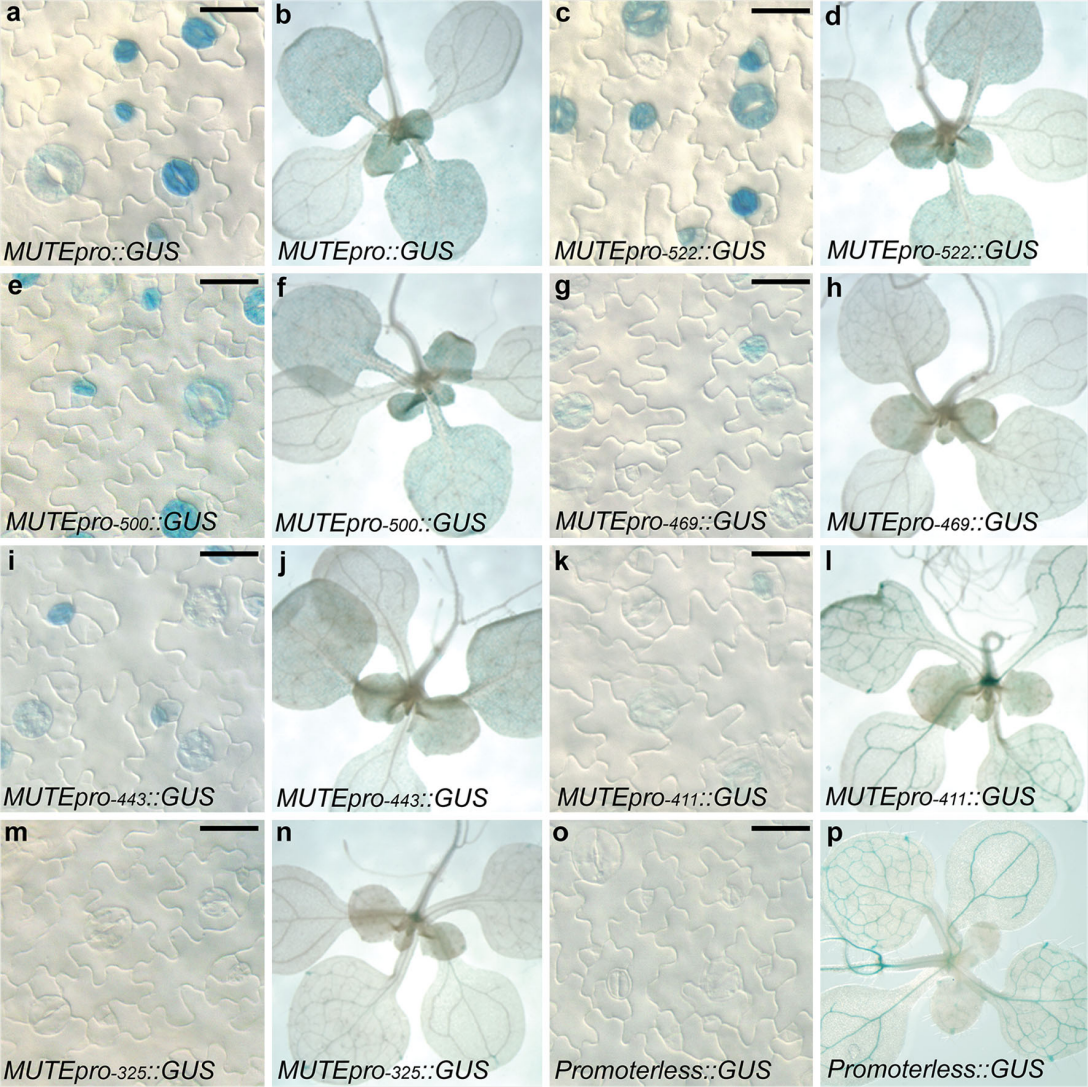
β-Glucoronidase (GUS) activity in transgenic lines. Successive deletions of the *MUTEpro*::*GUS* reporter constructs are indicated (**a**–**p**). Representative images of the abaxial leaf epidermis of 12-day-old seedlings (**a**, **c**, **e**, **g**, **i**, **k**, **m**, **o**) and whole-mount 14-day-old seedlings (**b**, **d**, **f**, **h**, **j**, **l**, **n**, **p**) are shown for each construct. No detectable qualitative difference in GUS expression was detected in promoters at least 500 bp in length compared to full-length (**a**–**f**). Consistent visible reduction in GUS expression was observed in promoters 469 bp or less in length (**g**–**n**). High background vascular and root expression was detected in promoterless::GUS plants, but no stomata-lineage expression was detected (**o**, **p**). *Scale bar* 10 µm for epidermal images

The activity of the full-length *MUTE* promoter (1.9 kb from the translational start site) has been analyzed previously and shown to be specific to the stomatal lineage and hydathode. No expression outside the stomatal lineage has been observed using the full-length promoter (Pillitteri et al. 2007, 2008). To gain insight into the functional role of putative *cis*-elements in the *MUTE* promoter, progressive 5′ promoter deletions were generated through PCR amplification and used to drive expression of the β-glucuronidase (GUS) reporter gene (Fig. 3, Suppl. Fig. S1). At least six independent transgenic lines were selected for histochemical analysis for each construct (Fig. 4). We detected high and consistent levels of ectopic reporter expression outside the stomatal lineage using small promoter fragments and promoterless constructs (Fig. 4l, n, p). This ectopic expression made quantitative fluorometric or PCR analysis on the reporter lines irrelevant. Therefore, we relied on the detectable qualitative changes within the stomatal lineage to delimit the *MUTE* 5′-regulatory region (Figs. 3, 4).

Using the full-length promoter, *MUTE* expression initiates in a subset of meristemoids and is observed only in stomatal-lineage cells in the epidermis (Fig. 4a). Using qualitative assessment, no observable difference in spatial or temporal expression of *MUTE* promoter activity was identified for promoters at least 500 bp in length compared to full-length (Fig. 4a–f, Suppl. Fig. S1). However, promoter fragments that were less than 500 bp in length resulted in a qualitative reduction and ultimately absence of detectable GUS expression in the stomatal lineage (Fig. 4g–n). Specifically, a consistent reduction in GUS activity was observed using 469 and 443 bp of the *MUTE* promoter compared to full-length (Fig. 4g–j). Further reduction in promoter size dramatically reduced activity, where 411 bp displayed minimal activity and 325 bp generated no detectable expression in the stomatal lineage, identical to the promoterless control plants (Fig. 4m–p). Thus, we identified a graduated decrease in expression with increasing loss of promoter sequence. We conclude that the minimal elements necessary for stomatal-lineage specific expression reside between 500 and 325 bp upstream of the translational start site of *MUTE*.

### 175 bp-region is sufficient to initiate meristemoid-specific expression

**Fig. 5:**
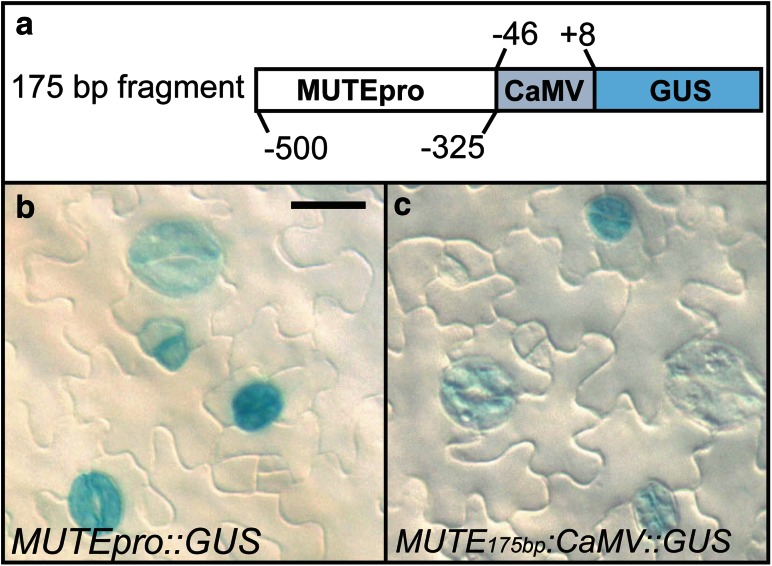
Regulatory region complementation. **a** Diagram of the complementation construct. The *MUTE* promoter fragment from −500 to −325 bp was fused to the 35S CaMV minimal promoter (−46 to +8 bp) (Oropeza-Aburto et al. 2012). **b**, **c** DIC image of the abaxial leaf epidermis from 12-day-old seedlings. Full-length *MUTE* promoter driving GUS expression (**b**) and complementation construct driving GUS expression (**c**). *Scale bar* 10 µm for epidermal images

Our deletion analysis revealed a short region from −500 to −325 bp that is important for *MUTE* promoter activity. To determine if this 175-bp region was sufficient to drive reporter gene expression, we joined one copy of the *MUTE* promoter fragment (−500 to −325 bp) to the 35S cauliflower mosaic virus (CaMV) minimal promoter (−46 to +8 bp relative to TTS) and analyzed its ability to drive GUS expression. This region of the CaMV promoter has been demonstrated to function as a core binding site (Oropeza-Aburto et al. 2012). The 175-bp chimeric promoter was sufficient to activate expression in the stomatal lineage, similar to the full-length promoter (Figs. 4a, 5), although expression intensity using the chimeric promoter was consistently weaker than full length. In addition to stomatal-lineage expression, we observed a low number of isolated patches of expression in vasculature tissue using the chimeric fragment that we do not observe using the native full-length promoter. This data indicates that key elements sufficient for proper spatiotemporal expression are present between −500 and −325 bp of the *MUTE* promoter.

To further dissect the functionality of this promoter fragment, we investigated a subregion of the 175-bp fragment (−411 to −325 bp) for its ability to initiate meristemoid-specific expression (Suppl. Fig. S2). This region was chosen based on our promoter deletion analysis, where the 411-bp promoter displayed very weak activity (Fig. 4k), suggesting that this region contained elements sufficient for basal expression. In contrast to the 175-bp fragment, none of the lines examined using the −411 to −325 bp fragment produced stomatal-lineage specific GUS expression (Suppl. Fig. S2). This smaller 86-bp region fused to the 35S CaMV minimal promoter was unable to mimic *MUTE* expression in the absence of the native 3′-flanking sequence. Taken together, these data suggests that the 89-bp region between −500 and −411 bp contains regulatory elements that are critical for robust *MUTE* expression. This is consistent with the notable loss of expression in our deletion experiments when the region from −500 to −411 bp is removed from the native promoter (Fig. 4g–l).

### Known *cis*-elements are not required for *MUTE* expression

**Fig. 6:**
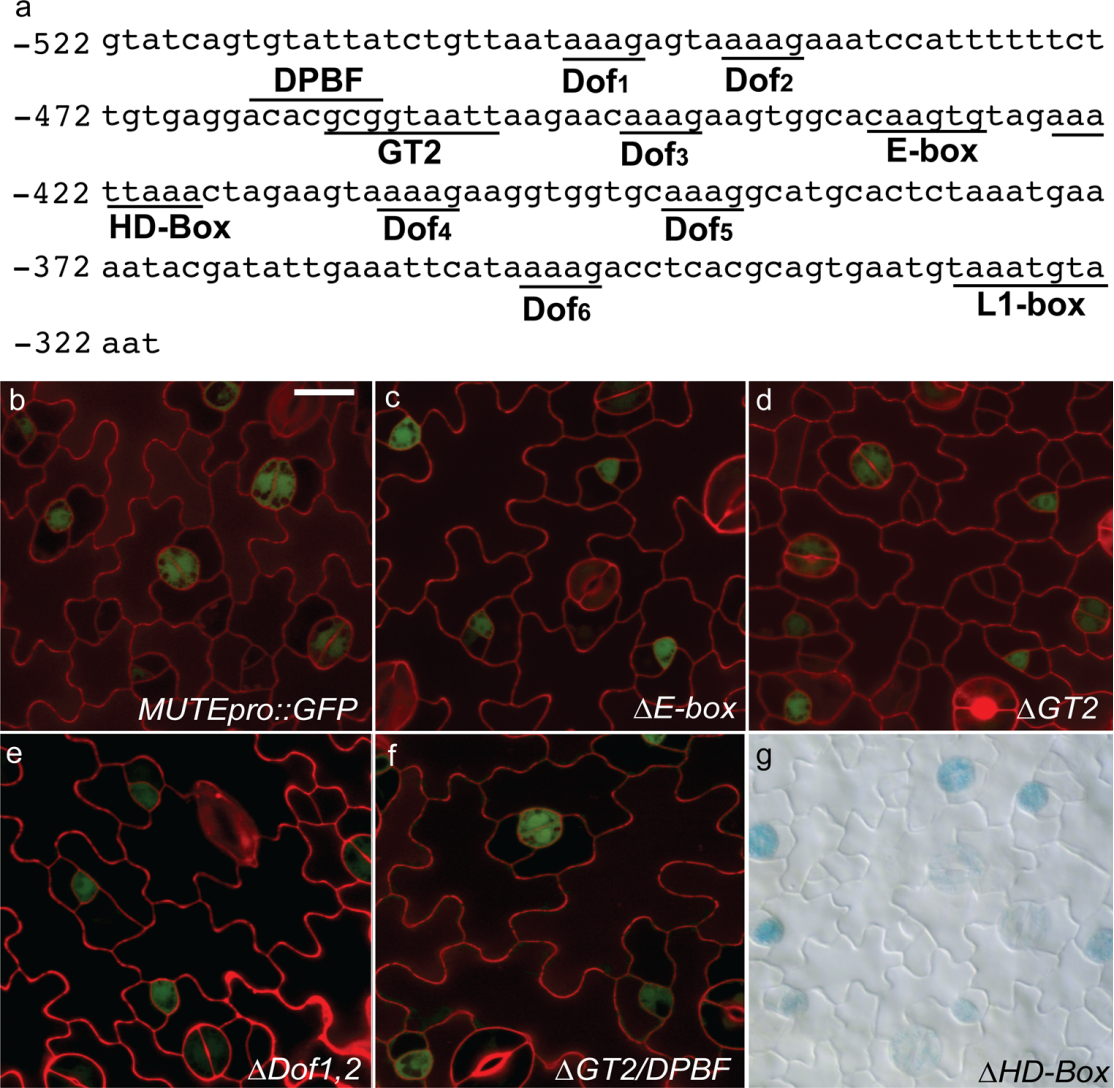
Site-directed mutagenesis of known *cis*-elements. **a** Diagram of the location of known *cis*-elements in the *MUTE* promoter fragment. Nucleotide location is given relative to the translational start site (+1). **b**–**g** Representative images of the abaxial leaf epidermis of 12-day-old seedlings. Confocal (**b**–**f**) images of GFP reporter constructs driven by mutagenized promoters as indicated. Cell borders were visualized using propidium iodide. DIC (**g**) image of mutagenized promoters driving the expression of GUS. See Suppl. Fig. S2 for specific nucleotide changes. *Scale bar* 10  µm for epidermal images

The promoter deletion and promoter sufficiency analysis suggested that elements between −500 and −325 bp were important for activity. Therefore, we identified potential *cis*-acting elements within this region of the *MUTE* promoter using publically available data (Table 1). *In silico* analysis of this promoter fragment showed that it contained 6 putative *cis*-elements including an E-box, [homeodomain-leucine zipper (HD-box), Dc3 promoter binding factor element (DPBF), GT2, L1-Box, and several Dof binding motifs (Table 1; Suppl. Fig. S2] (Dehesh et al. 1990; Abe et al. 2001; Kim et al. 2002; Davuluri et al. 2003; Bao et al. 2004; Yanagisawa 2004; Chang et al. 2008; Gordan et al. 2013). The DPBF and GT2 elements overlap (Fig. 6a).

**Table 1:** *Cis*-elements identified in the truncated *MUTE* promoter

*Cis*-Element	Sequence and position^a^	Function	Reference
DPBF	−465 ACACGCG	DPBF bZIP transcription factor binding core	Kim et al. (2002)
HD-box	−425 AAATTAAA	BELL1-like homeobox binding core	Bao et al. (2004)
GT2	−460 GCGGTAATT	GT-2 transcription factor binding core	Dehesh et al. (1990)
Dof	−499 AAAG	Dof transcription factor binding core	Yanagisawa (2004)
	−491 AAAG		
	−445 AAAG		
	−407 AAAG		
	−393 AAAG		
	−350 AAAG		
E-box	−433 CANNTG	bHLH transcription factor binding core	Gordan et al. (2013)
L1-box	−328 TAAATGYA^b^	Homeodomain leucine zipper binding core	Abe et al. (2001)

^a^Position is relative to the translation start site (+1)

^b^Y indicates a T or A at that position

To determine if these known *cis*-elements were specifically required for *MUTE* promoter activity, we performed site-directed mutagenesis to disrupt these elements in the context of a functional *MUTE* promoter (Suppl. Fig. S3). For this analysis we focused on elements in the region between −500 and −411 bp because they appear to be necessary for robust expression (Fig. 4e–j). Reporter constructs were produced by inserting the mutated promoter sequence upstream of either green fluorescent protein (GFP) or GUS. If any of these elements are important for *MUTE* expression, mutagenizing the sites should knock-down or eliminate reporter gene expression. Our data indicate that loss of these regulatory elements in the combinations used in this study did not decrease reporter gene expression (Fig. 6b–g). *MUTE* promoter activity with regard to intensity and spatiotemporal pattern was maintained across all constructs tested. This indicates that either a novel element not tested in this study is responsible for the stomatal-lineage specific expression of *MUTE* or that combinatorial control among multiple elements not tested in this study is required for proper expression.

### Comparison of *MUTE* promoters in the Brassicaceae

**Fig. 7:**
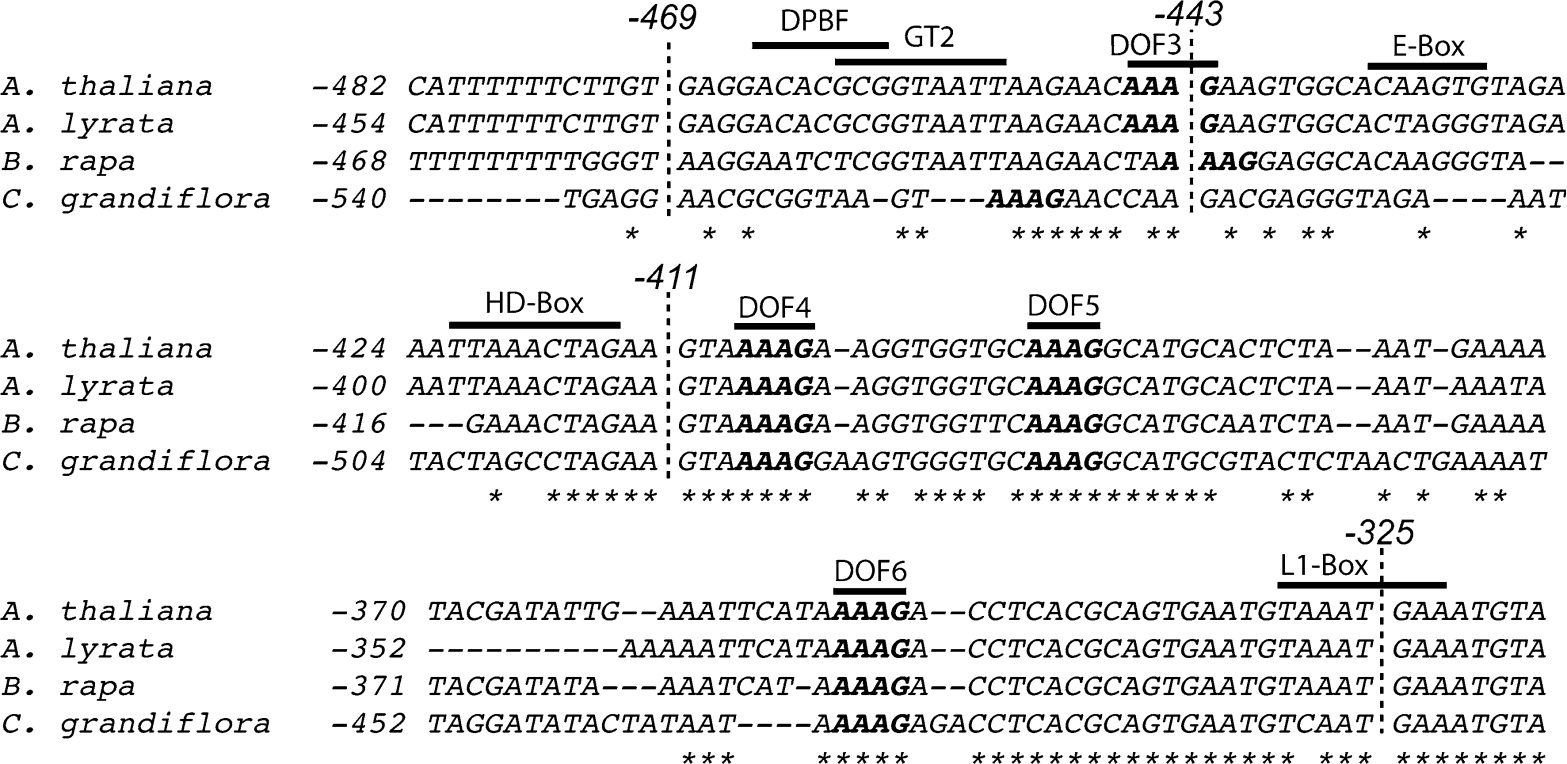
*MUTE* promoter comparison. Multiple sequence alignment of *MUTE* promoter fragments from *A. thaliana,* *A. lyrata, Brassica rapa,* and *Capsella grandiflora.* Nucleotide position is relative to the translational start site (+1). Known *cis*-elements are indicated. Conserved residues across all four species are indicated with an *asterisk*. Conserved locations of Dof elements (AAAG) are in *bold*. The location of the truncation points for promoter deletion constructs used in this study are indicated with a *dashed line*

To identify similarities in promoter structure among closely related members of the Brassicaceae, we aligned the fragment from *A. thalianaMUTE* promoter shown to be important for expression with those of orthologous promoters in *A. lyrata*, *Brassica rapa* and *Capsella grandiflora* (Fig. 7). The sequence used in our analysis was 92 % identical to *A. lyrata*, 84 % identical to *B. rapa* and 74 % identical to *C. grandiflora*, consistent with their phylogenic relationship. Of the specific known elements identified and tested in this study, only the Dof elements are spatially conserved among all promoter fragments, suggesting they may be important in regulation. The promoter region between −411 and −325 bp contains three conserved Dof elements, whereas the region between −469 and −411 bp contains one conserved Dof element (Fig. 7). In addition, an L1-box is conserved among three of the promoters. This element was not specifically tested here because a strong loss of promoter expression was detected even when the L1-box was intact (Fig. 4k). Although E-boxes were identified in the 1-kb sequence upstream of the start codon in all promoters, none of the related species had a conserved E-box element within the promoter region identified as important for *MUTE* expression in *A. thaliana*. In addition to the known elements, there are several conserved areas among all promoters that are not known binding sites for transcription factor or elements that affect transcription.

## Discussion

The bHLH protein MUTE triggers the transition from meristemoid to GMC, making it a critical regulator of the cellular decision to divide or differentiate. Master regulators that control important cellular transitions have been identified in many developmental contexts (Weintraub et al. 1991; Ghysen et al. 1993) and genome-scale analyses have provided information about potential regulatory networks responsible for differentiation of cell types (Pillitteri et al. 2011; Tripathi et al. 2014; Adrian et al. 2015; Kuenne et al. 2015). However, identification of specific regulatory sequences is limited using these approaches. Using the *MUTE* promoter, we generated transgenic *Arabidopsis* plants carrying reporter constructs driven by serial promoter deletions to identify important 5′-regulatory regions. Through progressive removal of regulatory sequence we identified a region of the *MUTE* promoter that is both necessary and sufficient for proper expression.

Our results indicated that *MUTE* promoter function markedly decreased in the absence of an 89-bp fragment between nucleotides −500 and −411 bp (Fig. 4). Although the native −411-bp promoter was able to activate transcription at a low level (Fig. 4k), this region was insufficient to drive expression in the context of the CaMV chimeric promoter (Suppl. Fig. S2). This data, together with the fact that the 175-bp fragment from −500 to −325 bp was sufficient to activate transcription suggests that important elements are located in the region between −500 and −411 bp. The less robust transcription from our chimeric promoter fragments may reflect the need for specific basal transcription factors at the minimal promoter, which has been shown to be important in the specific expression of several genes (Rabenstein et al. 1999; Butler and Kadonaga 2002).

A recent study by Lau et al. (2014) determined that SPCH binds the *MUTE* promoter at −1482 and −220 bp from the translational start site. Their work suggested a possible direct link between the bHLH transcription factors that promote stomata development. The homeodomain-leucine zipper protein, HOMEODOMAIN GLABROUS2 (HDG2), has also been shown to bind the *MUTE* promoter (Peterson et al. 2013). Specifically, HDG2 transcriptionally activated *MUTE* in a transient assay by binding to the L1-box. The data presented here do not support that those regions of the promoter are critical for *MUTE* expression, but our data do not exclude the possibility of combinatorial control where alternate binding sites outside of our 175-bp region may function to enhance *MUTE* expression or change expression under variable conditions.

Gene-specific transcriptional regulation is mediated by *cis*-acting elements that specify the site, timing and level of gene expression. Specific known elements between −500 and −411 bp, including Dof elements, E-box, HD-box, and a GT2 element were independently mutagenized and did not result in a detectable change in promoter activity (Fig. 6, Suppl. Fig. S3). This suggests that the regulatory elements that drive *MUTE* expression may work in combination and therefore loss of any single element does not disrupt expression to a detectable extent. Alternatively, an individual element not tested in study is responsible for *MUTE* expression. Although there are many documented cases of individual elements being responsible for the majority of gene expression (Ulmasov et al. 1997; Gomez-Porras et al. 2007; Oropeza-Aburto et al. 2012), the graduated reduction of expression in the *MUTE* promoter suggests combinatorial control may be important. Combinatorial regulation has been demonstrated for many genes and it may be particularly important for genes responding to multiple pathways. It has been well established that stomatal development is an adaptable trait that is controlled by many different inputs such as light, CO_2_, humidity, temperature, and constitutive developmental pathways (Gray et al. 2000; Lake and Woodward 2008; Kang et al. 2009; Casson and Hetherington 2010; Pillitteri and Torii 2012). Due to the critical importance of stomata and their plastic development, it is perhaps not surprising that a single element was not identified in this study.

Comparison of the *Arabidopsis* promoter sequence with those of orthologs revealed conserved regions, some of which did not include known *cis*-elements. Among known elements, only the clustered placement of Dof core binding motifs (AAAG) was conserved among all four orthologous promoters. Clusters of AAAG sites have been shown to additively contribute to guard cell-specificity of *AtMYB60* (Plesch et al. 2001; Cominelli et al. 2011). Our delineated area between −500 and −325 bp contains six Dof binding sites, higher than what would occur randomly in a sequence that size. Four of the Dof elements are conserved among close relatives. Our promoter truncation series removes sequential conserved Dof elements, therefore, our promoter comparison in combination with promoter deletion analysis suggests that Dof transcription factors may be important for *MUTE* regulation. We identified additional conserved sequences in the promoter that are not known to bind transcription factors, however, the absolute conservation among the orthologs may suggest some functional importance (Fig. 7). Overall, it is likely that *MUTE* is under the control of many loci, which activate or inhibit expression to fine-tune the production of stomata. With the identification of a promoter region important for *MUTE* expression, we have tools to investigate proteins bind and regulate *MUTE*, which ultimately contributes to mapping the genetic network controlling stomata production.

### *Author contribution statement*

LJP conceived and designed research. AM, EMA, RAB, AFW, JJF, KCS and LJP conducted experiments. LJP, AM and AW analyzed data. LJP wrote the manuscript. All authors read and approved the manuscript.

## Electronic supplementary material

Below is the link to the electronic supplementary material.

**Suppl.** **Fig.** **S1** Comparison of *MUTE* promoter activity. Confocal images of abaxial leaf epidermis. **a** *MUTE* full-length promoter driving green fluorescent protein (GFP). **b** 1305 bp of the *MUTE* promoter driving GFP. Promoter length is relative to the translational start site (+1). Cell borders are stained with propidium iodide. *Scale bar* 10  µm (TIFF 6131 kb)

**Suppl.** **Fig.** **S2** Regulatory region complementation. **a** Diagram of the complementation construct. The *MUTE* promoter fragment from −411 to −325 bp was fused to the 35S CaMV minimal promoter (−46 to +8 bp) (Oropeza-Aburto et al. 2012). **b**, **c** DIC image of the abaxial leaf epidermis from 12-day old seedlings. Full-length *MUTE* promoter driving GUS expression (**b**) and complementation construct driving GUS expression (**c**). *Scale bar* 10 µm for epidermal images (TIFF 8236 kb)

**Suppl.** **Fig.** **S3** Site-directed mutagenesis. **a** Sequence of the *MUTE* promoter fragment; location of specific elements are indicated. Nucleotide position is relative to translational start site (+1). **b**–**d** Changes made to designated elements are indicated. All nucleotide substitutions were made in the context of a functional >522-bp promoter (TIFF 12019 kb)

Supplementary material 4 (DOCX 40 kb)

Supplementary material 5 (DOCX 53 kb)

Supplementary material 6 (DOCX 63 kb)

Supplementary material 7 (DOCX 79 kb)

## Abbreviations

Dof: DNA binding with one finger
GUS: β-Glucoronidase
GMC: Guard mother cell
GFP: Green fluorescent protein
CaMV: Cauliflower mosaic virus
