# Supplementary figures and images for: Functional analysis of the Arabidopsis thalianaMUTE promoter reveals a regulatory region sufficient for stomatal-lineage expression
Source: Planta. 2016 Jan 9;243:987–98. doi: 10.1007/s00425-015-2445-7 (PMC4819751; doi:10.1007/s00425-015-2445-7)

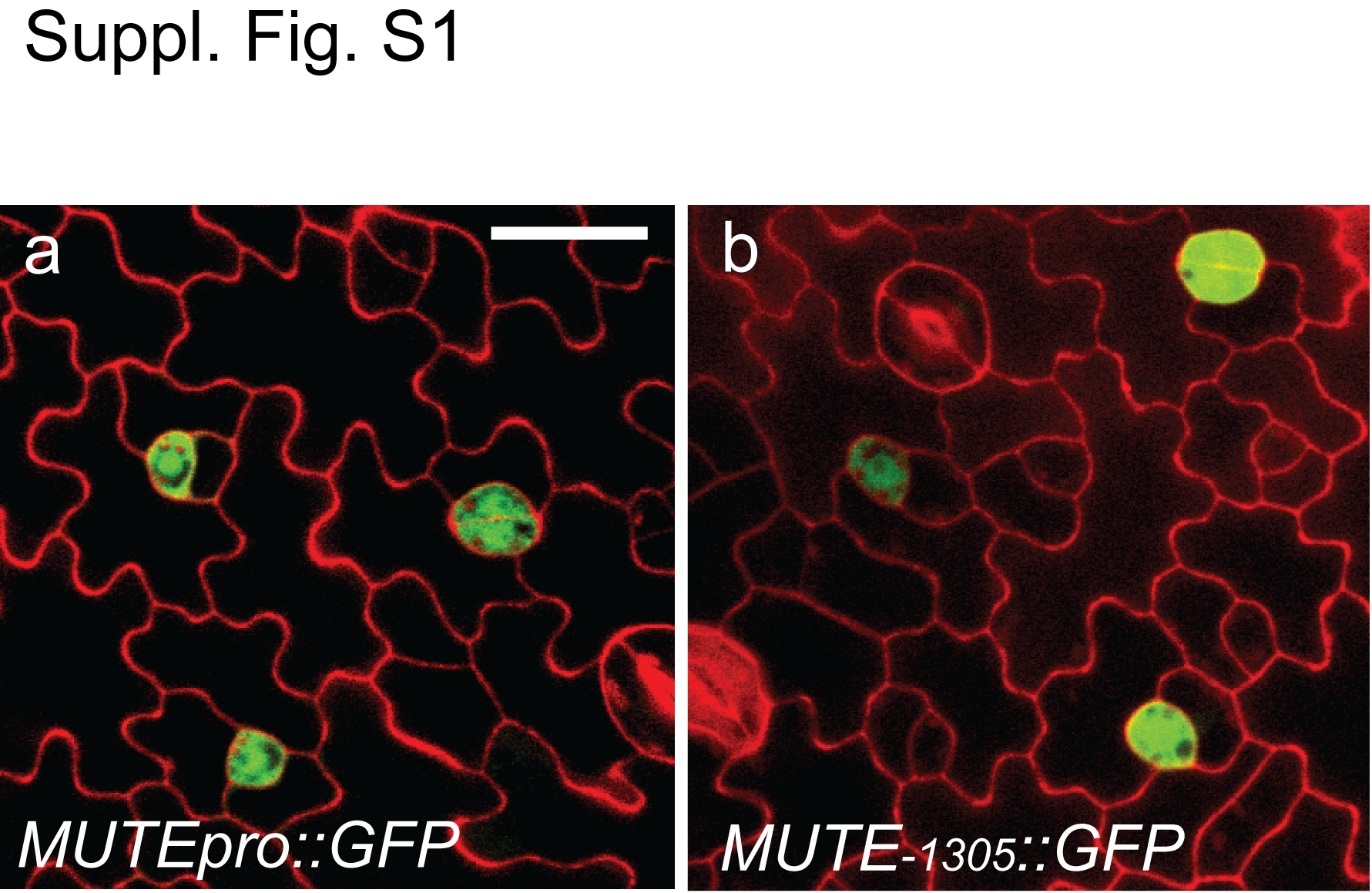

Supplement: Supplementary file 1 — Suppl. Fig. S1 Comparison of MUTE promoter activity. Confocal images of abaxial leaf epidermis. a MUTE full-length promoter driving green fluorescent protein (GFP). b 1305 bp of the MUTE promoter driving GFP. Promoter length is relative to the translational start site (+1). Cell borders are stained with propidium iodide. Scale bar 10 µm (TIFF 6131 kb) [file 425_2015_2445_MOESM1_ESM.tif]

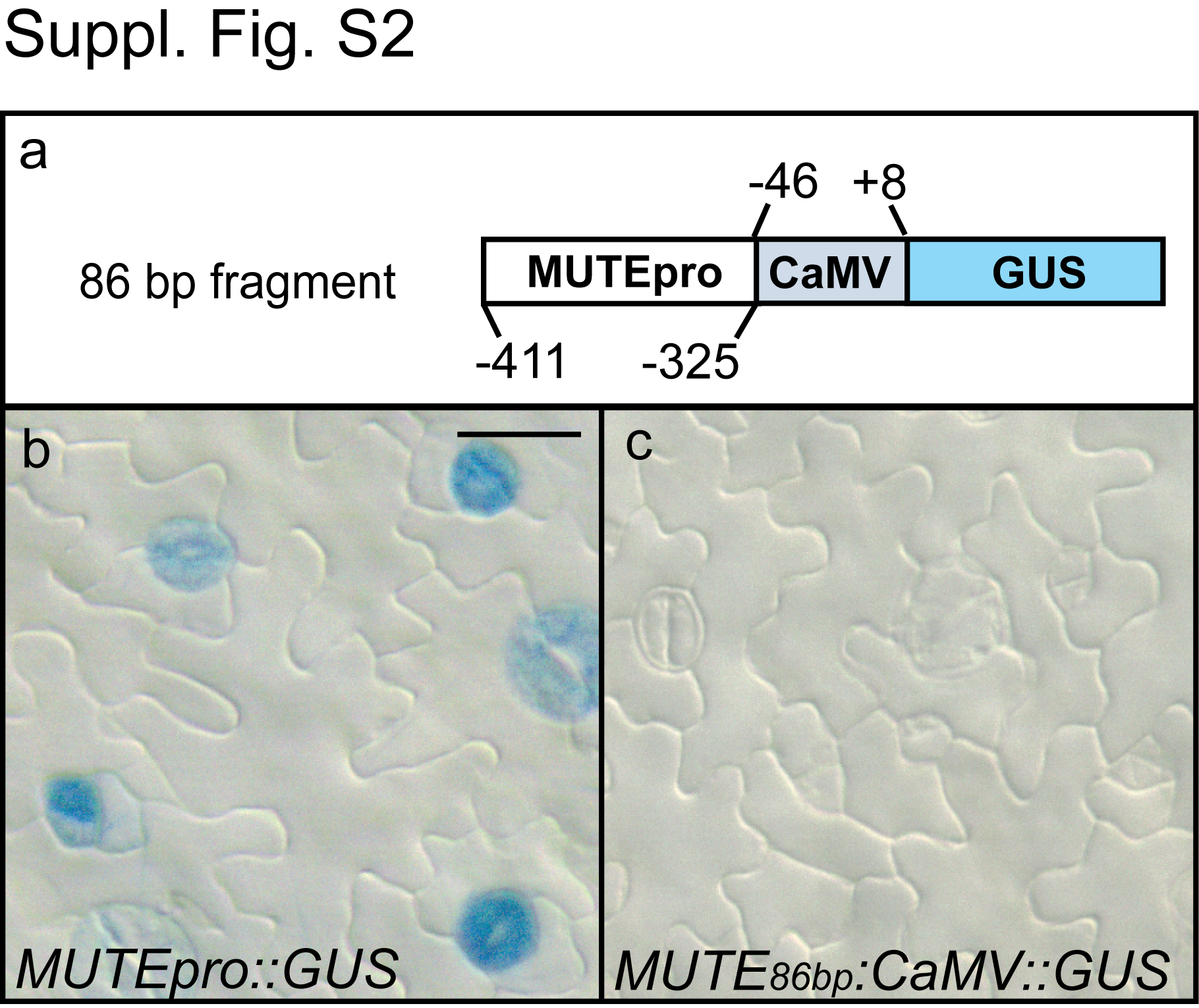

Supplement: Supplementary file 2 — Suppl. Fig. S2 Regulatory region complementation. a Diagram of the complementation construct. The MUTE promoter fragment from −411 to −325 bp was fused to the 35S CaMV minimal promoter (−46 to +8 bp) (Oropeza-Aburto et al. 2012). b, c DIC image of the abaxial leaf epidermis from 12-day old seedlings. Full-length MUTE promoter driving GUS expression (b) and complementation construct driving GUS expression (c). Scale bar 10 µm for epidermal images (TIFF 8236 kb) [file 425_2015_2445_MOESM2_ESM.tif]

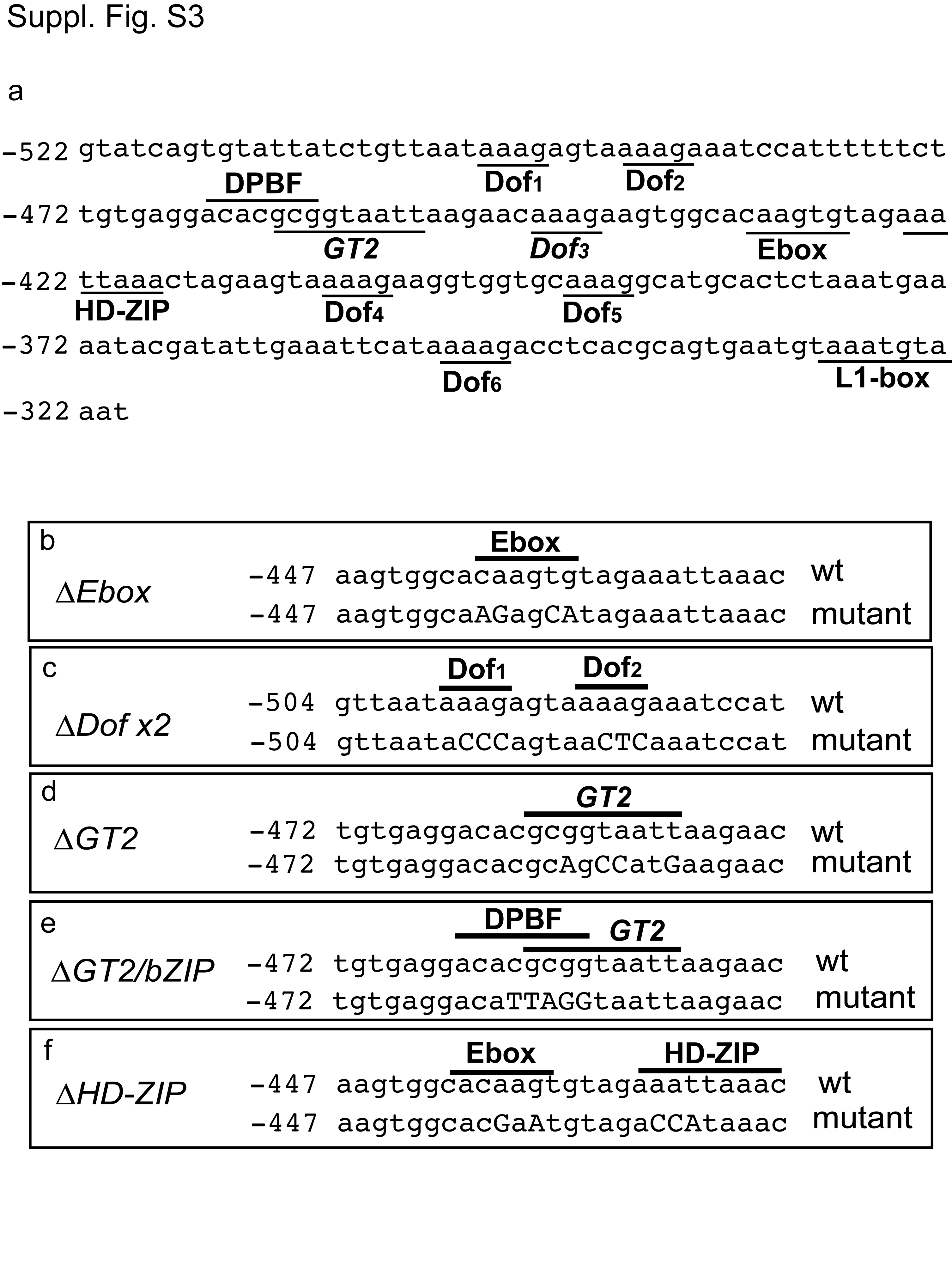

Supplement: Supplementary file 3 — Suppl. Fig. S3 Site-directed mutagenesis. a Sequence of the MUTE promoter fragment; location of specific elements are indicated. Nucleotide position is relative to translational start site (+1). b–d Changes made to designated elements are indicated. All nucleotide substitutions were made in the context of a functional >522-bp promoter (TIFF 12019 kb) [file 425_2015_2445_MOESM3_ESM.tif]
