# Supplementary material for: Functional analysis of the Arabidopsis thalianaMUTE promoter reveals a regulatory region sufficient for stomatal-lineage expression
Source: Planta. 2016 Jan 9;243:987–98. doi: 10.1007/s00425-015-2445-7 (PMC4819751; doi:10.1007/s00425-015-2445-7)
Supplement: Supplementary file 4 — Supplementary material 4 (DOCX 40 kb) [file 425_2015_2445_MOESM4_ESM.docx]

**Suppl. Table S1** *MUTE*-specific sequence of oligonucleotides used in 5’ and 3’ RACE

| Primer name | Sequence^a^ |
| --- | --- |
| MUTE3'RACE767 | TAGGAGCCGCCACTACCCGAGTACCG |
| MUTE3'RACE913 | GTCTCTAGGCGAATCGTGGGGCAGCTCG |
| MUTE5'RACE941RC | CGAGCTGCCCCACGATTCGCCTAGAGAC |
| MUTE5'RACE793RC | CGGTACTCGGGTAGTGGCGGCTCCTA |

^a^ Sequence given 5’ to 3’
