# Supplementary material for: Functional analysis of the Arabidopsis thalianaMUTE promoter reveals a regulatory region sufficient for stomatal-lineage expression
Source: Planta. 2016 Jan 9;243:987–98. doi: 10.1007/s00425-015-2445-7 (PMC4819751; doi:10.1007/s00425-015-2445-7)
Supplement: Supplementary file 5 — Supplementary material 5 (DOCX 53 kb) [file 425_2015_2445_MOESM5_ESM.docx]

**Suppl.Table S2** Sequence of oligonucleotides used to produce deletion derivatives of the

*MUTE* promoter

| Primer name | Sequence^a^ |
| --- | --- |
| MUTE-1305GW | CACCCATAGAAGTGTTATCCAAG |
| MUTE-522ECO | CGGAATTCGTATCAGTGTATTATCTGTT |
| MUTE-500ECO | CGGAATTCTAAAGAGTAAAAGAAATCCATTTTTTC |
| MUTE-469ECO | CGGAATTCGAGGACACGCGGTAATTAAG |
| MUTE-443ECO | CGGAATTCAAGAAGTGGCACAAGTGTAG |
| MUTE-411ECO | CGGAATTCGTAAAAGAAGGTGGTGCAAAGG |
| MUTE-325ECO | CGAATTCGTAAATGTATCCAACTTCAC |
| MUTE-1ECO | CGGAATTCGATACTTAATTGATCAAGATTC |

^a^ Sequence given 5’ to 3’
