# Supplementary material for: Functional analysis of the Arabidopsis thalianaMUTE promoter reveals a regulatory region sufficient for stomatal-lineage expression
Source: Planta. 2016 Jan 9;243:987–98. doi: 10.1007/s00425-015-2445-7 (PMC4819751; doi:10.1007/s00425-015-2445-7)
Supplement: Supplementary file 6 — Supplementary material 6 (DOCX 63 kb) [file 425_2015_2445_MOESM6_ESM.docx]

**Suppl. Table S3** Sequence of oligonucleotides used to produce complementation constructs

| Oligo name | Sequence^a^ |
| --- | --- |
| MUTEXHO-500 | CTCGAGTAAAGAGTAAAAGAAATCC |
| MUTEBH1-325.RC | GGATCCCATTTACATTCACTGCGTG |
| MUTE-500ECO | GCCGAATTCTAAAGAGTAAAAGAAATCCAT |
| 35SMINECO.RC | GCCCGAATTCCCCTCCTCTCCAAATGAAATG |
| MUTE MINPRO R | AATTCCCTCCTCTCCAAATGAAATGAACTTCCTTATATAGAGGAAGGGT  CTTGATTTACATTCACTGCGTGAGGTCTTTTATGAATTTCAATATCGTAT  TTTCATTTAGAGTCGATGCCTTTGCACCACCTTCTTTTACG |
| MUTE MINPRO F | AATTCGTAAAAGAAGGTGGTGCAAAGGCATGCACTCTAAATGAAAATACGATATTGAAATTCATAAAAGACCTCACGCAGTGAATGTAAATCAAGACCCTTCCTCTATATAAGGAAGTTCATTTCATTTGGAGAGGAGGG |

^a^ Sequence given 5’ to 3’
