# Supplementary material for: Functional analysis of the Arabidopsis thalianaMUTE promoter reveals a regulatory region sufficient for stomatal-lineage expression
Source: Planta. 2016 Jan 9;243:987–98. doi: 10.1007/s00425-015-2445-7 (PMC4819751; doi:10.1007/s00425-015-2445-7)
Supplement: Supplementary file 7 — Supplementary material 7 (DOCX 79 kb) [file 425_2015_2445_MOESM7_ESM.docx]

**Suppl. Table 4** Primers used to produce site-directed mutagenesis constructs

| Primer name | Affected Site | Sequence^a^ |
| --- | --- | --- |
| Standard end primers |  |  |
| MUTE-1219GW |  | CACCGTAATATGAATTGAGATATTGTT |
| MUTE-1RC |  | GATACTTAATTGATCAAGATTC |
|  |  |  |
| Internal mutant primers |  |  |
| MUTE-504 | Dof1/2 | GTTAATACCCAGTAACTCAAATCCATTTTT |
| MUTE-504RC | Dof1/2 | AAAAATGGATTTGAGTTACTGGGTATTAAC |
| MUTE-472 | DPBF/GT2 | TGTGAGGACATTAGGTAATT |
| MUTE-472RC | DPBF/GT2 | AATTACCTAATGTCCTCACA |
| MUTE-433 | Ebox | GAAGTGGCACGAATGTAGACCATAAACTAG |
| MUTE-433RC | Ebox | CTAGTTTATGGTCTACATTCGTGCCACTTC |
| MUTE-428 | HD-ZIP | tagtaccattgctagaagtaaaagaaggtg |
| MUTE-428RC | HD-ZIP | CAATGGTACTACACTTGTGCCACTTCTT |
| MUTE-507M^b^ | GT2 | TATCTGTTAATAAAGAGTAAAAGAAATCCATTTTTTCTTGTGAGGACACGCAGCCATGAAG |

^a^ Sequence given 5’ to 3’

**^b^** The GT2 mutant version of the promoter is in the context of a 507 bp promoter established in this study to function as the full-length promoter. All other mutant versions of the promoter are in the context of a 1219 bp promoter.
